# The potential impact of reduced international donor funding on the household economic burden of tuberculosis in low- and middle-income countries: A modeling study

**DOI:** 10.1371/journal.pmed.1004946

**Published:** 2026-02-20

**Authors:** Allison Portnoy, Rebecca A. Clark, Mark Jit, Christopher Finn McQuaid, Alexandra S. Richards, Roel Bakker, Tom Sumner, Tomos O. Prŷs-Jones, Rein M. G. J. Houben, Richard G. White, Katherine C. Horton, Nicolas A. Menzies

**Affiliations:** 1 Department of Global Health, Boston University School of Public Health, Boston, Massachusetts, United States of America; 2 Center for Health Decision Science, Harvard T.H. Chan School of Public Health, Boston, Massachusetts, United States of America; 3 TB Modelling Group, London School of Hygiene and Tropical Medicine, London, United Kingdom; 4 Centre for Mathematical Modelling of Infectious Diseases, London School of Hygiene and Tropical Medicine, London, United Kingdom; 5 Department of Infectious Disease Epidemiology, London School of Hygiene and Tropical Medicine, London, United Kingdom; 6 Department of Global and Environmental Health, New York University School of Global Public Health, New York, United States of America; 7 School of Health and Wellbeing, University of Glasgow, Glasgow, United Kingdom; 8 Department of Global Health and Population, Harvard T.H. Chan School of Public Health, Boston, Massachusetts, United States of America; McGill University, CANADA

## Abstract

**Background:**

Recent shifts in the global health funding landscape—most notably the dismantling of the United States Agency for International Development (USAID) and possible reduced contributions to the Global Fund to Fight AIDS, TB, and Malaria (Global Fund)—threaten essential tuberculosis (TB) services in low- and middle-income countries (LMICs). We quantified the potential impact on the household economic burden of TB.

**Methods and findings:**

We used linked epidemiological and economic models, calibrated to 79 LMICs, to estimate future TB patient costs under six scenarios: continuation of 2024 funding levels (baseline), termination of USAID, termination of USAID plus announced reductions in Global Fund contributions from the USA alone, termination of USAID plus complete termination of Global Fund contributions from the USA alone, termination of USAID plus announced reductions in Global Fund contributions from all donor countries contributing 1% or more to the budget, and full elimination of external funding for TB. Outcomes included total TB-attributable household costs and numbers of households experiencing catastrophic costs (disease-related costs >20% of annual income). USAID termination was projected to produce US$7.5 (95% uncertainty interval: $6.1–8.9) billion in additional patient-incurred costs and 3.9 (3.1–4.6) million additional households experiencing catastrophic costs over 2025–2050. The worst-case scenario (elimination of all external funding) resulted in $79.7 ($60.0–99.2) billion in additional patient-incurred costs and 40.5 (30.9–50.7) million additional households experiencing catastrophic costs—a 32% increase over baseline. Impacts were greatest for poorer households, with over 50% of additional catastrophic costs occurring in the poorest 20% of households. This analysis is limited by substantial uncertainty regarding costs faced by untreated patients and assumptions of constant patient costs and uniform treatment reductions over time.

**Conclusions:**

Abrupt reductions in international donor funding for TB may reverse recent progress toward financial risk protection and health equity in LMICs. Strategies to reduce the disruption caused by funding cuts and protect vulnerable populations are urgently needed.

## Author summary

**Why was this study done?:**

- Tuberculosis can result in substantial medical costs and income loss that can threaten financial security for affected families in low- and middle-income countries (LMICs).
- International donor funding has played a major role in reducing both cases of tuberculosis and the associated financial burden on households, but recent policy changes have put this funding at risk.
- This study estimated how reductions in donor funding could affect the economic burden of tuberculosis on patients, especially among the poorest households.

**What did the researchers do and find?:**

- We adapted a system of linked epidemiological and economic models covering 79 LMICs to estimate tuberculosis-affected household costs under several scenarios of reduced international funding between 2025 and 2050.
- Ending funding for tuberculosis testing and treatment from the United States Agency for International Development alone was estimated to result in an additional 7.5 billion US dollars in patient costs and nearly 4 million more households experiencing catastrophic costs.
- In the most severe scenario, eliminating all external funding for tuberculosis could lead to nearly 80 billion US dollars in additional household costs and more than 40 million extra households facing catastrophic costs, with the largest impacts falling on the poorest households.

**What do these findings mean?:**

- Large and sudden reductions in international funding for tuberculosis could reverse progress in protecting families from severe financial hardship, particularly among the poorest populations.
- The findings highlight the importance of maintaining stable financing for tuberculosis testing and treatment services or developing alternative funding strategies to protect vulnerable households.
- This analysis is limited by substantial uncertainty regarding costs faced by untreated patients and assumptions of constant patient costs and uniform treatment reductions over time.

## Abstract 3

In a modelling study, Allison Portnoy and colleagues investigate the potential impact of reduced international donor funding on the household economic burden of Tuberculosis in low- and middle-income countries.Image: https://pixabay.com/illustrations/debt-money-credit-loan-mortgage-3272735/Credit: Tumisu via Pixabay

## Introduction

Tuberculosis (TB) is the world’s leading cause of death from an infectious agent, responsible for an estimated 1.25 million deaths in 2023 [1]. Despite slow declines in TB incidence since 2000, global rates have been increasing since 2020—approximately 10.8 million individuals are estimated to have developed TB in 2023, up from 10.1 million in 2020 [1]. The impact of this morbidity and mortality falls disproportionately on low- and middle-income countries (LMICs), with 30 LMICs with the highest TB burden accounting for 87% of overall incidence [1,2].

The costs incurred by individuals who develop TB place a high financial burden on TB-affected households in LMIC settings. These costs arise from the out-of-pocket expenses (such as transport and accommodation) required to participate in TB treatment, as well as the income losses incurred by individuals who are unable to work. Due to these two factors, more than 50% of TB-affected households face total costs during the TB episode that are greater than 20% of annual household income, and therefore categorized as catastrophic costs by the World Health Organization (WHO) [1]. These impacts are greater for TB-affected households in the poorest income quintile, with 75% of these households incurring catastrophic costs [3]. Reducing the costs faced by patients and their households is a high-level objective of the WHO End TB Strategy, yet progress to eliminate catastrophic costs due to TB has been slow [4].

International donor funding has played an important role historically in supporting access to TB prevention and treatment services, which contribute to reducing household economic burden in resource-limited settings. Until early 2025, the primary international funders of the global TB response were the United States Agency for International Development (USAID), which provided 19% of international donor funding reported by national TB programs (NTPs) in 2023, and the Global Fund to Fight AIDS, TB, and Malaria (Global Fund), which provided 76% of international donor funding—38% contributed by the United States of America—in the same year [1,5]. The financial support of these agencies, led by the largest single country donor (the United States of America), enabled progress towards closing the diagnostic and treatment gaps faced by people with TB and their households, and mitigating the high financial costs faced by TB patients.

Recent progress in combating TB is now threatened by changes to the international donor funding landscape. In early 2025, the United States abruptly dismantled USAID, canceling bilateral health aid provided by the agency [6]. The ramifications of this loss of funding have already interrupted TB prevention, diagnostic, and treatment services [7,8]. Budget proposals within the U.S. government in July 2025 proposed substantially reduced support for international health programs, including the Global Fund. In addition to the United States, other donor countries have announced reductions in contributions to bilateral aid programs and multinational organizations [6].

In this study, we examined how changes in donor funding could impact the economic burden of TB on affected households in LMICs as defined by 2025 World Bank income group [9]. To do so, we extended an earlier study that projected the potential health impact of funding reductions [10] to quantify the household economic consequences of TB, the number of TB-affected households experiencing catastrophic costs, and the distribution of these outcomes across income quintiles in each country over the period 2025–2050. We conducted this analysis for 79 LMICs—representing 91% of TB incidence in LMICs.

## Methods

### Scenarios

We examined five scenarios describing different patterns of future funding for the TB response in the 79 LMICs included in the analysis, including 66 eligible for funding from the Global Fund [10]. First, we simulated a scenario representing the impact of the termination of USAID funding to NTPs beginning in 2025. Second, we simulated a scenario representing both the termination of USAID and announced reductions in funding to the Global Fund from the United States alone, equivalent to a 13% reduction in total Global Fund contributions. Third, we simulated a scenario representing both the termination of USAID and a complete termination of United States’ funding to the Global Fund, equivalent to a 17% reduction in total Global Fund contributions. Fourth, we simulated a scenario representing the impact of both the termination of USAID funding and announced reductions in funding to the Global Fund, equivalent to a 46% reduction in total Global Fund contributions. Finally, we examined a scenario estimating the impact of complete termination of both USAID and Global Fund funding, as well as termination of external TB funding from other non-government and private organizations. Each scenario was compared with a baseline scenario assuming TB funding and programmatic activities continue at 2024 levels.

We assumed all funding cuts would be introduced in 2025, sustained into the future, and not replaced by other funding sources, and that, unless specified in a scenario, other funding sources (domestic, other international) would continue at 2024 levels (additional details reported in Clark and colleagues) [10]. We assumed the impact of reduced funding for TB programs could be represented by reductions in access to TB diagnosis and treatment. These reductions represent decreased tuberculosis screening and active case finding, diagnosis (including rapid testing, drug susceptibility testing, specimen transport, and other diagnostic processes), contact investigation, community-supported tuberculosis care and stigma reduction, procurement and supply chains for diagnostics, external technical assistance, and capacity building and training [8]. We operationalized this by assuming there would be a reduction in the TB treatment initiation rate (for individuals with untreated TB disease) that is proportional to the budget reduction represented by a given scenario. These budget components were estimated from expenditure data reported by countries to WHO, which has monitored funding for TB programs since 2022 [1]. For each country, WHO data were used to calculate the proportion of total expected funding from all sources from USAID, from the Global Fund, and from other external donors in 2023.

### Mathematical model

To undertake the analysis, we adapted a system of linked epidemiological and economic models previously used for other policy evaluations (in particular, the implications of global TB vaccine introduction) [11–13]. Models were calibrated to demographic, epidemiological, and health service data for 79 LMICs [10]. For each country, calibration targets included country-specific tuberculosis incidence rates, country-specific tuberculosis notification rates, country-specific tuberculosis mortality rates, and the global proportion of asymptomatic tuberculosis episodes among infectious tuberculosis episodes. Calibration was performed using history matching with emulation, as implemented by the *hmer* package in R [14] and Approximate Bayesian Computation sampling, generating 200 fitted parameter sets per country. We used the epidemiological model to project health impact through 2050 under each scenario, as compared to the baseline scenario defined above (Box A and Figs A and B in S1 Appendix). Additional details are provided in Clark and colleagues [10,11].

### Costs incurred by TB-affected households

For each modeled country, we disaggregated the TB burden projected under each scenario by income quintile, based on survey data describing differences in the relative prevalence of TB by household income [13]. To calculate the costs incurred by TB patients and their households we multiplied the simulated number of TB cases (by country, income quintile, scenario, and year) by the average patient cost per TB episode. Estimates of the patient costs per TB episode, stratified by country and household income quintile, were derived from a published meta-regression analysis of 22 nationally representative TB patient cost surveys [3], including 14 from high TB burden settings [15]. These estimates included direct medical costs (medical products and services), direct non-medical costs (patient out-of-pocket costs for travel, accommodation, food, and nutritional supplements) and indirect costs (income losses) incurred by TB patients. For each country and income quintile, we assumed that the per-patient costs of TB (in 2021 constant dollars) would not change in future years. For the base-case analysis, we assumed that individuals with TB disease who do not receive appropriate treatment (i.e., received ineffective care, or not treated at all) experience the same total per episode costs as those who received appropriate treatment. We examined alternative assumptions in sensitivity analyses.

### Catastrophic costs

As defined in WHO End TB Strategy targets, we calculated catastrophic costs as instances where the patient costs of TB disease—the sum of direct medical costs, direct non-medical costs and indirect costs—exceeded 20% of total annual income for the TB-affected household [16–19]. For each country and income quintile, we assessed the number of TB-affected households experiencing catastrophic costs under each scenario, multiplying the probability of catastrophic costs per TB episode by the simulated number of TB cases by country, income quintile, scenario, and year. Estimates of the probability of catastrophic costs per TB episode (stratified by country and income quintile) were derived from the meta-analysis of TB patient cost surveys used for patient cost estimates [3]. For each country and income quintile, we assumed that the probability of catastrophic costs for TB patients would not change in future years.

### Distribution of benefits across countries and income strata

We undertook additional analyses to describe how the number of households experiencing catastrophic costs was distributed across the collective income gradient of the modeled countries. First, we ordered all country income quintiles (79 countries × 5 quintiles = 395 unique groups) by average per capita gross domestic product (GDP) in 2021 purchasing power parity (PPP)-adjusted dollars. To do so, we obtained estimates of per capita PPP GDP and the fraction of total income held by each country income quintile, imputing missing values according to WHO region and income level group averages (e.g., low-income countries in the African region) [9,20]. We multiplied these two terms and divided by the population fraction in each quintile (0.2) to obtain the average per capita PPP GDP for each quintile. We ranked all quintiles by average per capita PPP GDP and calculated the distribution of each study outcome across these quintiles. We summarized results graphically as well as via the Concentration Index, which quantifies the relative concentration of a given outcome in high-income or low-income groups. For a cumulative distribution representing incidence of an outcome in percentiles of a population ordered from poorest to richest (the ‘Concentration Curve’), the Concentration Index is defined as two times the area between the Concentration Curve and the line of equality (the 45° line, representing an equal distribution of the outcome across income groups). The index is defined in [−1, 1], with more positive (negative) values indicating greater concentration of the outcome in higher (lower)-income groups [21].

### Sensitivity analysis

Key parameters used in the analysis are summarized in Table A in S1 Appendix. We propagated uncertainty in analytic inputs using a second-order Monte Carlo simulation, which generated 200 estimates for each outcome to reflect economic and epidemiological parameter uncertainty, including uncertainty in data used to fit the epidemiological model. We used this distribution of estimates to generate equal-tailed 95% uncertainty intervals for each study outcome [22]. We also examined the robustness of results to alternative analytic assumptions. First, as there is substantial uncertainty around the costs incurred by patients who do not receive appropriate TB treatment, we re-estimated results under alternative scenarios that assumed costs for this group were 50% lower and higher, respectively, compared with appropriately treated individuals (versus the main analysis which assumed treated and untreated patients bore the same costs). Second, we examined alternative thresholds for defining catastrophic costs as 10% and 25% of annual household income (versus 20% in the main analysis). We did not include an examination of structural model uncertainty.

## Results

### Overall potential impact of funding cuts

Compared with the baseline scenario, the termination of USAID funding in the 79 modeled countries was projected to result in $7.5 (95% uncertainty interval: $6.1–8.9) billion in additional costs borne by TB-affected households, including $1.2 ($0.8–1.5) billion in direct medical costs, $2.8 ($2.1–3.4) billion in direct non-medical costs, and $3.6 ($2.8–4.6) billion in indirect costs over the 2025–2050 study period (Table 1). For this scenario, we estimated 3.9 (3.1–4.6) million additional households would experience catastrophic costs, as compared to the baseline. If the United States alone were to reduce its contributions from the Global Fund, it could result in $22.6 ($18.1–26.9) billion in increased costs borne by TB-affected households, whereas complete termination of all contributions from the United States alone could result in $24.0 ($19.2–28.5) billion in increased costs. Reductions in funding to the Global Fund from all donor countries contributing 1% or more to the budget in addition to the termination of USAID could result in approximately $28.7 ($22.7–34.4) billion in increased costs to TB-affected households, and an additional 14.6 (11.6–17.6) million households experiencing catastrophic costs due to TB (a 12% increase compared to baseline).

**Table 1:** Costs borne by TB-affected households and number of households with catastrophic costs.

Scenario	Increased patient direct medical costs (billions)	Increased patient direct non–medical costs (billions)	Increased patient indirect costs (billions)	Increased patient total costs (billions)	Increased cases of catastrophic costs (millions)
Termination of USAID	1.2 (0.8–1.5)	2.8 (2.1–3.4)	3.6 (2.8–4.6)	7.5 (6.1–8.9)	3.9 (3.2–4.6)
	*2.3% (1.7%–3.0%)*	*2.3% (1.7%–2.9%)*	*2.7% (2.2%–3.2%)*	*2.5% (2.0%–2.9%)*	*3.1% (2.5%–3.6%)*
Termination of USAID and reduced Global Fund contributions from the USA	3.6 (2.5–4.8)	8.5 (6.2–10.6)	10.5 (8.0–13.3)	22.6 (18.1–26.9)	11.5 (9.2–13.6)
	*7.2% (5.3%–9.3%)*	*6.9% (5.0%–8.9%)*	*7.9% (6.4%–9.4%)*	*7.4% (5.7%–8.9%)*	*9.1% (7.2%–11.0%)*
Termination of USAID and termination of Global Fund contributions from the USA	3.9 (2.7–5.1)	9.0 (6.6–11.3)	11.1 (8.5–14.1)	24.0 (19.2–28.5)	12.2 (9.8–14.5)
	*7.7% (5.7%–9.9%)*	*7.4% (5.3%–9.4%)*	*8.3% (6.7%–10.0%)*	*7.8% (6.1%–9.5%)*	*9.6% (7.6%–11.7%)*
Termination of USAID and reduced Global Fund contributions from donor countries contributing 1% or more to the budget	4.6 (3.2–6.1)	10.8 (7.8–13.6)	13.2 (10.0–16.8)	28.7 (22.7–34.4)	14.6 (11.6–17.6)
	*9.2% (6.7%–11.9%)*	*8.8% (6.3%–11.3%)*	*9.9% (8.0%–12.0%)*	*9.4% (7.2%–11.5%)*	*11.5% (9.0%–14.1%)*
Termination of all external TB funding	13.2 (8.7–18.1)	30.1 (20.7–39.5)	36.4 (26.4–47.3)	79.7 (60.0–99.2)	40.5 (30.9–50.7)
	*26.3% (19.4%–34.4%)*	*24.6% (16.5%–33.4%)*	*27.3% (20.8%–35.0%)*	*26.0% (19.2%–33.8%)*	*31.9% (24.2%–41.3%)*

Estimates include 79 low- and middle-income countries analyzed compared to the baseline scenario. Values in italicized text represent percentage changes compared to the baseline scenario. Values in parentheses represent equal-tailed 95% credible intervals. Total costs included patient direct medical, direct non-medical, and indirect costs (all undiscounted) over 2025–2,050 in 2021 US dollars. Catastrophic costs are defined as instances where the total patient costs incurred during an episode of TB disease exceeded 20% of total annual household income. GF,  the Global Fund to Fight AIDS, TB, and Malaria; TB,  tuberculosis; USAID, United States Agency of International Development.

If all external TB funding were terminated, we projected an increase of $79.7 ($60.0–99.2) billion in total patient costs, including $13.2 ($8.7–18.1) billion in direct medical costs, $30.1 ($20.7–39.5) billion in direct non-medical costs, and $36.4 ($26.4–47.3) billion in indirect costs. This would result in an estimated 40.5 (30.9–50.7) million additional cases of households experiencing catastrophic costs, an increase of nearly 32% compared to what was projected under the baseline. We visualized the range of estimated impact by country and scenario, comparing the reduction in funding to each country to the percentage increase in the number of households experiencing catastrophic costs over the analytic period (Fig C in S1 Appendix).

### Distribution of TB patient costs within each country

Across all modeled countries and scenarios, the wealthiest two income quintiles accounted for approximately 51% of increased total patient costs resulting from cuts to international donor funding (Concentration Index 0.14 for each scenario), with greater costs per episode of TB care incurred in these groups outweighing the greater burden of TB cases in poorer quintiles.

**Fig 1:**
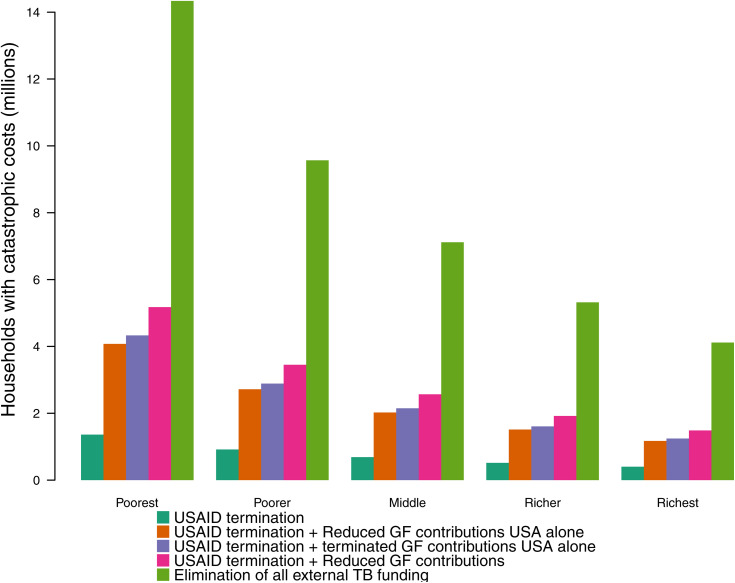
Number of increased tuberculosis (TB)-affected households experiencing catastrophic costs by within-country income quintile, compared to no changes in donor funding. Total costs borne by TB-affected households are categorized as ‘catastrophic’ if they exceed 20% of total household’s annual income. The total cost of a TB episode included patient direct medical, direct non-medical and indirect costs over 2025–2050. GF, the Global Fund to Fight AIDS, TB, and Malaria; TB, tuberculosis; USAID, United States Agency of International Development.

**Fig 2:**
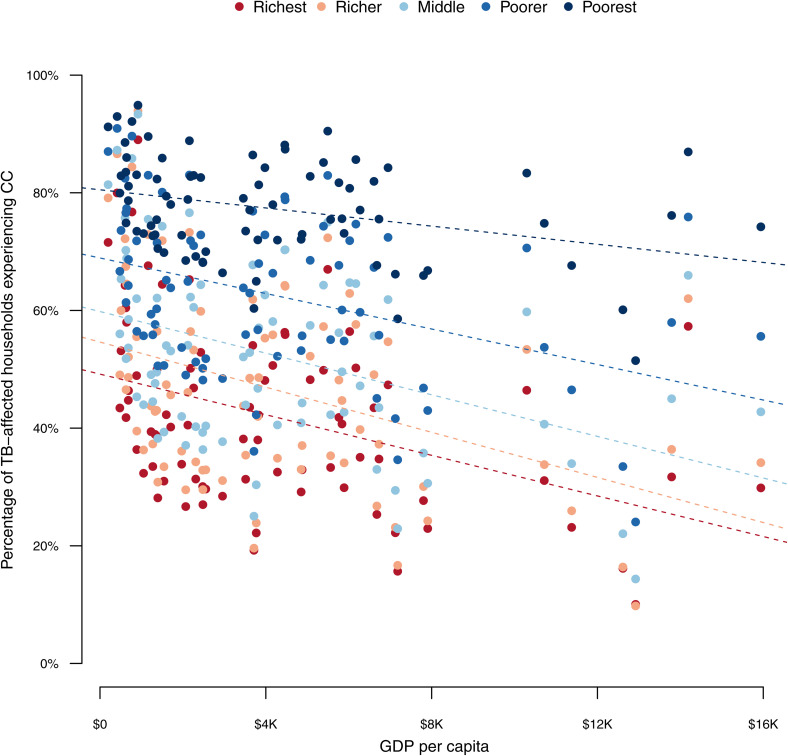
Percentage of tuberculosis (TB)-affected households experiencing catastrophic costs (CC) by gross domestic product (GDP) per capita and by income quintile in 79 low- and middle-income countries. Dotted lines represent trends for the percentage of TB-affected households experiencing catastrophic costs by GDP per capita for each income quintile. Colors represent income quintiles for each analyzed country. CC, catastrophic costs; GDP, gross domestic product; TB, tuberculosis.

The largest absolute increases in the proportion of households facing catastrophic costs were in lower income quintiles within each country. Under each scenario, nearly 60% of cases of catastrophic costs were experienced by the poorest two quintiles (Concentration Index −0.24 for each scenario). Fig 1 shows the relative magnitude of cases of catastrophic costs averted across income quintiles by analytic scenario. The percentage of TB-affected households experiencing catastrophic costs by country GDP per capita and by income quintile is presented in Fig 2, demonstrating the increasing magnitude of additional cases of catastrophic costs experienced with decreasing household income.

### Distribution of outcomes across countries and income strata

**Fig 3:**
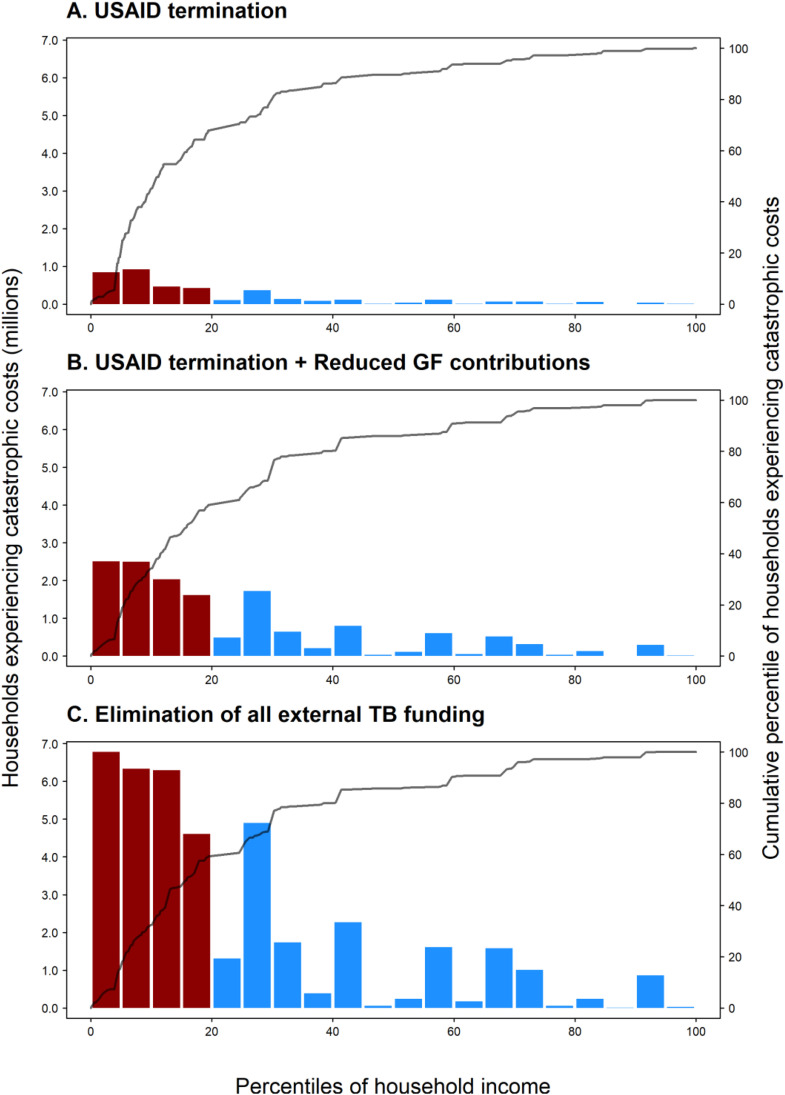
Distribution of the number of TB-affected households experiencing catastrophic costs over 2025–2050 under alternative scenarios for possible reductions in external TB funding, across all modeled strata, ordered by household income. Bars defined by left-hand side y-axis; lines defined by right-hand side y-axis. Ordering of population by household income based on average 2,021 per capita GDP in purchasing power parity (PPP) dollars, for each modeled stratum (395 total strata). Bars shaded red indicate the poorest 20% of modeled population by PPP GDP per capita; bars shaded blue indicate the upper 80% of the modeled population. GDP, gross domestic product; GF, the Global Fund to Fight AIDS, TB, and Malaria; TB, tuberculosis; USAID, United States Agency of International Development.

Fig 3 shows the distribution of TB-affected households experiencing catastrophic costs by household income across the combined population of the 79 modeled LMICs over the 2025–2050 period for the three of the five scenarios (additional scenarios provided in Fig D in S1 Appendix), each compared to a counterfactual scenario without funding losses. In all scenarios, expected increases to the number of households experiencing catastrophic costs were concentrated in poorer households (poorest quintile shaded in red), ranging from 59% (Concentration Index −0.53) to 68% (Concentration Index −0.61) of the total cases of catastrophic costs in the poorest 20% of households across analyzed scenarios.

### Sensitivity analyses

Assuming that individuals with TB who do not receive appropriate treatment experience 50% lower costs compared with treated individuals reduced the number of households with catastrophic costs for each scenario (Table B in S1 Appendix).

Assuming that individuals with TB who do not receive appropriate treatment experience 50% higher costs compared with treated individuals increased the number of households with catastrophic costs for each scenario (Table C in S1 Appendix).

Defining catastrophic costs as instances where patient costs exceed 10% of annual household income (versus 20% in the main analysis), resulted in approximately 39% more cases of catastrophic costs, as compared to the main analysis (Table D in S1 Appendix). Defining catastrophic costs as instances where patient costs exceed 25% of annual household income resulted in approximately 14% fewer cases of catastrophic costs compared to the main analysis.

## Discussion

This study quantifies the potential impact of reduced health aid by the United States and other countries on the costs faced by TB-affected households in LMICs. In a worst-case scenario of the full termination of external TB funding, the results of this analysis suggest that TB-affected households could face $79.7 ($60.0–99.2) billion in additional direct medical, non-medical, and indirect costs for TB care, resulting in an estimated 40.5 (30.9–50.7) million additional households experiencing catastrophic costs. The magnitude of these impacts can be compared to a prior analysis estimating the potential health gains from a novel TB vaccine over 2028–2050 [13]. This earlier analysis estimated that new vaccine introduction could avert US$38 to US$44 billion in patient-incurred costs, with 23 million fewer households facing catastrophic costs due to TB. Based on this comparison, the potential negative consequences of reduced international donor funding for TB are almost double the potential benefits from a novel TB vaccine.

Sharp reductions in international donor funding pose significant threats to the continuity and quality of essential health services in LMICs. While external funding has played a transformative role in scaling up TB prevention and treatment, the abrupt change in the funding landscape since the beginning of 2025 has already led to service disruptions [6–8]. The withdrawal of donor support could erode healthcare access, particularly for the poorest households that experience the greatest burden of TB [13]. The timeline to achieve the End TB Strategy target to eliminate household catastrophic costs could also be substantially lengthened [4]. This study demonstrates that in addition to the health losses that could result from funding reductions [10], the projected economic burden that households will face given a sharp withdrawal of funding without alternative financing mechanisms could be catastrophic.

For the sustainability of NTPs, blended financing models that combine public, private, and donor resources may offer interim solutions, particularly in countries with constrained fiscal space, as countries work towards strengthening domestic resource mobilization. Additionally, there may be opportunities to integrate aspects of NTPs into primary healthcare in order to promote efficiency and enhance system resilience.

This analysis has several limitations. First, the 22 countries included in the previously published meta-regression analysis of TB patient costs were not a representative sample of all LMICs [3], but do include 14 high TB burden settings as defined by WHO [15]. Second, there is substantial uncertainty around the costs faced by individuals with TB who are not able to access treatment. While these individuals will incur substantial costs associated with care-seeking that does not end in appropriate treatment, as well as income losses from untreated TB morbidity, there is little empirical evidence quantifying the magnitude of these costs. In the main analysis, we assumed that untreated individuals experienced the same costs as treated individuals, based on limited evidence highlighting costs faced by these individuals [23]. We then examined alternative assumptions for the costs faced by untreated individuals as well as alternative thresholds for defining catastrophic costs as a percentage of total annual household income in sensitivity analysis. Third, we assumed that the current estimated costs of each episode for TB patients would not change over the 2025–2050 period (in constant US dollars). If the patient costs per TB episode were to decline, or real incomes rise, this would reduce the number of households experiencing catastrophic costs due to TB. Additionally, we did not consider that delayed diagnosis (as a consequence of funding cuts) could lead to prolonged or less successful treatment, due to more advanced disease at the time of treatment initiation. This could potentially increase the costs to patients. We also assumed that these costs will be borne by TB-affected households, but costs may in fact be too high to be borne, potentially resulting in increased TB mortality. Fourth, we assumed a standard reduction in treatment initiation for all TB patients in the underlying epidemiological modeling informing this analysis, but the impact on treatment initiation could differ based on patient characteristics, including household income. Furthermore, we did not consider potential impacts on drug-resistant TB resulting from interrupted or partial TB treatment resulting from funding cuts. Fifth, we did not consider the impact of funding cuts on TB preventive therapy (TPT) provision or the secondary effects of international donor funding cuts to other sectors or to other non-governmental providers of TB prevention, diagnosis, and treatment. The long-term effects of funding cuts (e.g., to nutrition, HIV programs) could further impact the number of households with TB and/or the number of households at risk of catastrophic costs due to TB-associated care. Moreover, as we have not previously seen funding interruptions at such a wide scale, the range of possible consequences is quite broad. Finally, the funding scenarios we examined were deliberately simplified to reveal the potential impact of current and possible funding cuts. It is unlikely that the trajectory of external health funding for the modeled countries will exactly match one of the scenarios examined in this analysis, but these scenarios represent extremes describing the possible range of consequences.

The findings of this study can contribute to the ongoing policy debates surrounding the risks and opportunities inherent in the shift from externally financed to domestically supported health systems. As described by Pai and colleagues (2024) [24], donors should support transition processes through flexible, phased approaches that allow for country-specific adaptation and a fair share of overhead costs. Global health agencies can play a facilitative role by providing technical assistance and capacity strengthening for partner institutions in LMICs. For LMIC governments, it is essential to invest in institutional capacity and enhance coordination across sectors and levels of government. Ultimately, a shared commitment to equity, sustainability, and health system strengthening must not only guide the transition toward more domestically anchored and resilient health systems, but also ensure due consideration to protect vulnerable populations in the world’s poorest countries in the face of reduced global health resources expected in coming years.

## Supporting information

S1 Appendix The potential impact of reduced international donor funding on the household economic burden of tuberculosis in low- and middle-income countries: a modeling study. (DOCX)

## Abbreviations

GDP: gross domestic product
LMICs: low- and middle-income countries
NTPs: national TB programs
PPP: purchasing power parity
TB: tuberculosis
USAID: United States Agency for International Development
WHO: World Health Organization
