# Supplementary material for: The potential impact of reduced international donor funding on the household economic burden of tuberculosis in low- and middle-income countries: A modeling study
Source: PLoS Med. 2026 Feb 20;23(2):e1004946. doi: 10.1371/journal.pmed.1004946 (PMC12948071; doi:10.1371/journal.pmed.1004946)
Supplement: S1 Appendix — (DOCX) [file pmed.1004946.s001.docx]

**S1 Appendix for: “The potential impact of reduced international donor funding on the household economic burden of tuberculosis in low- and middle-income countries: a modelling study”**

**Table of Contents**

[Supplementary Box A. Epidemiological model overview. 2](#_Toc221518029)

[Supplementary Figure A. Tuberculosis natural history model. 3](#_Toc221518030)

[Supplementary Figure B. Tuberculosis natural history model incorporating vaccination. 4](#_Toc221518031)

[Supplementary Table A. Sources and values of analytic inputs. 5](#_Toc221518032)

[Supplementary Figure C. Percentage increase in number of cases of catastrophic costs (CC), compared to baseline, compared to the reduction in international donor funding by country and scenario. 7](#_Toc221518033)

[Supplementary Figure D. Distribution of the number of TB-affected households experiencing catastrophic costs over 2025–2050 given (1) the termination of funding from USAID + reductions in contributions to GF from the USA alone; and (2) the termination of funding from USAID + termination of all contributions to GF from the USA alone, across all modelled strata, ordered by household income. 8](#_Toc221518034)

[Supplementary Table B. Costs borne by TB-affected households and number of households with catastrophic costs (in millions), assuming costs for untreated cases are 0.5x treated cases. 9](#_Toc221518035)

[Supplementary Table C. Costs borne by TB-affected households and number of households with catastrophic costs (in millions), assuming costs for untreated cases are 1.5x treated cases. 10](#_Toc221518036)

[Supplementary Table D. Number of households with catastrophic costs (in millions), assuming thresholds of 10%, 20% (base-case), and 25%. 11](#_Toc221518037)

# Supplementary Box A. Epidemiological model overview.

The core natural history model is specified in Supplementary Figure 1.

Those with no previous exposure or infection with *Mycobacterium tuberculosis* (*Mtb*) [Uninfected-Naive ($U_{N}$)] could become infected at rate $\lambda_{j}$ and progress to an Infection-Fast ($I_{F}$) class following initial infection. From Infection-Fast, three possible pathways were possible: (i) Fast progression to Subclinical Disease ($D_{S}$), where individuals are infectious with a reduced infectiousness compared to clinical tuberculosis, but display no symptoms of tuberculosis disease; (ii) self-clearance to Uninfected-Cleared ($U_{C}$), where individuals are no longer infected with *Mtb* and therefore are not at risk of progression to tuberculosis disease without reinfection; or (iii) continue to remain latently infected with a risk of reactivation and progression to disease, albeit at a lower rate than Infection-Fast, by transitioning to the Infection-Slow ($I_{S}$) class. Those in the Infection-Slow class could self-clear to the Uninfected-Cleared class, be reinfected and return to the Infection-Fast class, or reactivate their infection and progress to Subclinical Disease.

Once in the Subclinical Disease class, individuals could naturally cure (without treatment) to the Resolved ($R$) class, or progress to Clinical Disease ($D_{C}$), where individuals are infectious and display symptoms of tuberculosis disease. Treatment initiation from Clinical Disease to On-Treatment ($T$) began in 1960 and increased following a sigmoid curve to 2019, with average treatment duration assumed to be 6 months. Treatment completions transitioned to the Resolved class and treatment non-completions returned to Clinical Disease. Deaths occurring on-treatment and in clinical disease counted toward the total number of tuberculosis deaths during the year. Those with clinical disease could also naturally cure to the resolved class. Individuals in the Resolved class could be reinfected or relapse to Subclinical Disease but could not enter Infection-Fast or Infection-Slow directly. We assumed that the infection and resolved classes are partially protected against reinfection. In those who have self-cleared, we assumed the level of protection against reinfection is half of the protection against reinfection for the infection and resolved classes.

Age was modelled in single years from ages 0 to 79 and aggregated into two categories for ages 80 to

89, and ages 90 to 99. Births and ageing occurred at the beginning of each year.

Vaccines are incorporated in the tuberculosis natural history structure as indicated with the orange boxes in Supplementary Figure 2 by reducing the rate of progression to disease parameters into the subclinical disease compartment from the infection-fast, infection-slow, and resolved compartments by (1-p_V_), where p_V_ is the vaccine efficacy. Vaccine efficacy was modelled as “degree”, also known as “leaky”. Degree vaccines assume that everyone who has been vaccinated receives some protection from the vaccine equivalent to the value of the vaccine efficacy.

We independently calibrated one model for each country by identifying areas of the parameter space that made the output of each country-specific model match the corresponding calibration targets (historical epidemiological data). The model was fitted to calibration targets using history matching with emulation, a relatively new calibration method that allows us to explore high-dimensional parameter spaces efficiently and robustly. History matching progresses as a series of iterations, called waves, where implausible areas of the parameter space, i.e., areas that are unable to give a match between the model output (e.g., the predicted incidence rate by the model) and the empirical data (e.g., the incidence rate calibration target from data), are found and discarded. In order to identify implausible parameter sets, emulators are used. Emulators are statistical approximations of model outputs that are built using a modest number of model runs. Emulators provide an estimate of the value of the model at any parameter set of interest, with the advantage that they are orders of magnitude faster than the model.

History matching with emulation, implemented through the hmer package in R, considerably reduced the size of the parameter space to investigate. Rejection sampling was then performed on the reduced space to identify at least 200 parameter sets that matched all targets for each country.

Reproduced from: Clark RA, Mukandavire C, Portnoy A, Weerasuriya CK, Deol A, Scarponi D, et al. The impact of alternative delivery strategies for novel tuberculosis vaccines in low-income and middle-income countries: a modelling study. Lancet Glob Health. 2023;11(4):e546-e55. doi: 10.1016/s2214-109x(23)00045-1. PubMed PMID: 36925175; PubMed Central PMCID: PMCPMC10030455.

# Supplementary Figure A. Tuberculosis natural history model.


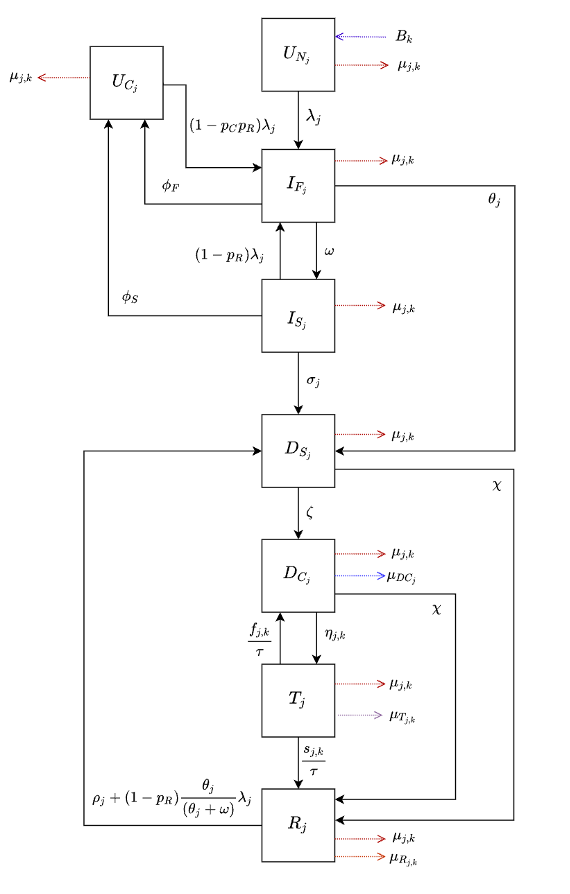


Abbreviations: DC = Clinical Disease; DS = Subclinical Disease; IF = Infection-Fast; IS = Infection-Slow; R = Resolved; T = On-Treatment; UC = Uninfected-Cleared; UN = Uninfected-Naive. Subscript *j* represents parameters that vary by age, and subscript k represents parameters that vary over time.

Reproduced from: Clark RA, Mukandavire C, Portnoy A, Weerasuriya CK, Deol A, Scarponi D, et al. The impact of alternative delivery strategies for novel tuberculosis vaccines in low-income and middle-income countries: a modelling study. Lancet Glob Health. 2023;11(4):e546-e55. doi: 10.1016/s2214-109x(23)00045-1. PubMed PMID: 36925175; PubMed Central PMCID: PMCPMC10030455.

# Supplementary Figure B. Tuberculosis natural history model incorporating vaccination.


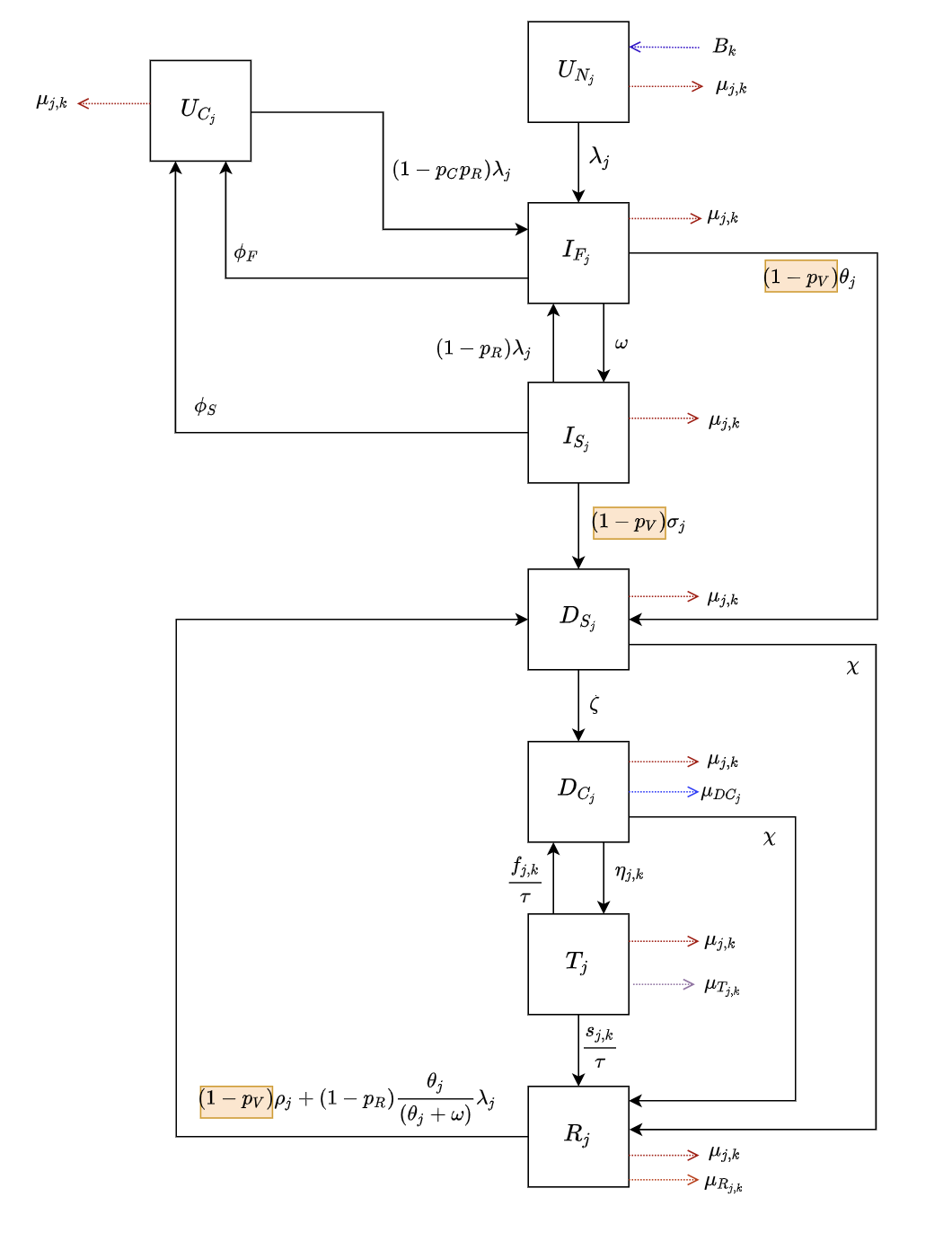


Abbreviations: DC = Clinical Disease; DS = Subclinical Disease; IF = Infection-Fast; IS = Infection-Slow; R = Resolved; T = On-Treatment; UC = Uninfected-Cleared; UN = Uninfected-Naive. Subscript *j* represents parameters that vary by age, and subscript k represents parameters that vary over time.

Reproduced from: Clark RA, Mukandavire C, Portnoy A, Weerasuriya CK, Deol A, Scarponi D, et al. The impact of alternative delivery strategies for novel tuberculosis vaccines in low-income and middle-income countries: a modelling study. Lancet Glob Health. 2023;11(4):e546-e55. doi: 10.1016/s2214-109x(23)00045-1. PubMed PMID: 36925175; PubMed Central PMCID: PMCPMC10030455.

# Supplementary Table A. Sources and values of analytic inputs.

| Input | Value/Assumption/Formula | Source |
| --- | --- | --- |
| **Total costs borne by TB-affected households, per episode** | Stratified by country, income quintile, and cost category | [1] |
| **Fraction of TB cases incurring catastrophic costs** | Stratified by country and income quintile | [1] |
| **Risk ratio of TB disease across income strata** | Poorest quintile: Reference  Poorer quintile: 0.8479  Middle quintile: 0.7477  Richer quintile: 0.6221  Richest quintile: 0.5157 | Quantitative synthesis of TB prevalence surveys [2–12] |
| **Total costs borne by TB-affected households that are not accessing care/ with untreated TB, compared to those accessing treatment** | 100% (50% and 150% in sensitivity analyses) | Assumption |
| **Discount rate** | Health outcomes: 0%  Cost outcomes: 0% | [13] |

Note: TB = tuberculosis.

1. Portnoy A, Yamanaka T, Nguhiu P, Nishikiori N, Floyd K, Garcia Baena I, Menzies NA. Costs incurred by people receiving TB treatment in low- and middle-income countries: a meta-regression analysis. The Lancet Global Health. 2023 Oct;11(10):e1640-e1647.
2. Harling G, Ehrlich R, Myer L. The social epidemiology of tuberculosis in South Africa: a multilevel analysis. Soc Sci Med. 2008;66(2):492-505. https://doi.org/10.1016/j.socscimed.2007.08.026
3. Hoa NB, Tiemersma EW, Sy DN, Nhung NV, Gebhard A, Borgdorff MW, et al. Household expenditure and tuberculosis prevalence in VietNam: prediction by a set of household indicators. Int J Tuberc Lung Dis. 2011;15(1):32-7.
4. Hossain S, Quaiyum MA, Zaman K, Banu S, Husain MA, Islam MA, et al. Socio economic position in TB prevalence and access to services: results from a population prevalence survey and a facility-based survey in Bangladesh. PLoS One. 2012;7(9):e44980. https://doi.org/10.1371/journal.pone.0044980
5. Kapata N, Chanda-Kapata P, Ngosa W, Metitiri M, Klinkenberg E, Kalisvaart N, et al. The Prevalence of Tuberculosis in Zambia: Results from the First National TB Prevalence Survey, 2013-2014. PLoS One. 2016;11(1):e0146392. https://doi.org/10.1371/journal.pone.0146392
6. Migambi P, Gasana M, Uwizeye CB, Kamanzi E, Ndahindwa V, Kalisvaart N, et al. Prevalence of tuberculosis in Rwanda: Results of the first nationwide survey in 2012 yielded important lessons for TB control. PLoS One. 2020;15(4):e0231372. https://doi.org/10.1371/journal.pone.0231372
7. Oxlade O, Murray M. Tuberculosis and poverty: why are the poor at greater risk in India? PLoS One. 2012;7(11):e47533. https://doi.org/10.1371/journal.pone.0047533
8. Singh SK, Kashyap GC, Puri P. Potential effect of household environment on prevalence of tuberculosis in India: evidence from the recent round of a cross-sectional survey. BMC Pulm Med. 2018;18(1):66. https://doi.org/10.1186/s12890-018-0627-3
9. Siroka A, Law I, Macinko J, Floyd K, Banda RP, Hoa NB, et al. The effect of household poverty on tuberculosis. Int J Tuberc Lung Dis. 2016;20(12):1603-8. https://doi.org/10.5588/ijtld.16.0386
10. van Leth F, Guilatco RS, Hossain S, Van't Hoog AH, Hoa NB, van der Werf MJ, et al. Measuring socio-economic data in tuberculosis prevalence surveys. Int J Tuberc Lung Dis. 2011;15 Suppl 2:58-63. https://doi.org/10.5588/ijtld.10.0417
11. Yates TA, Ayles H, Leacy FP, Schaap A, Boccia D, Beyers N, et al. Socio-economic gradients in prevalent tuberculosis in Zambia and the Western Cape of South Africa. Trop Med Int Health. 2018;23(4):375-90. https://doi.org/10.1111/tmi.13038
12. Foster N, Nguyen HV, Nguyen NV, Nguyen HB, Tiemersma EW, Cobelens FGJ, et al. Social determinants of the changing tuberculosis prevalence in Việt Nam: Analysis of population-level cross-sectional studies. PLoS Med. 2022;19(3):e1003935. https://doi.org/10.1371/journal.pmed.1003935
13. Vassall A, Sweeney S, Kahn J, Gomez GB, Bollinger L, Marseille E, et al. Global Health Costing Consortium reference case for estimating the costs of global health services and interventions. 2017. https://ghcosting.org/pages/standards/reference_case. (accessed 29 July 2022).

# Supplementary Figure C. Percentage increase in number of cases of catastrophic costs (CC), compared to baseline, compared to the reduction in international donor funding by country and scenario.


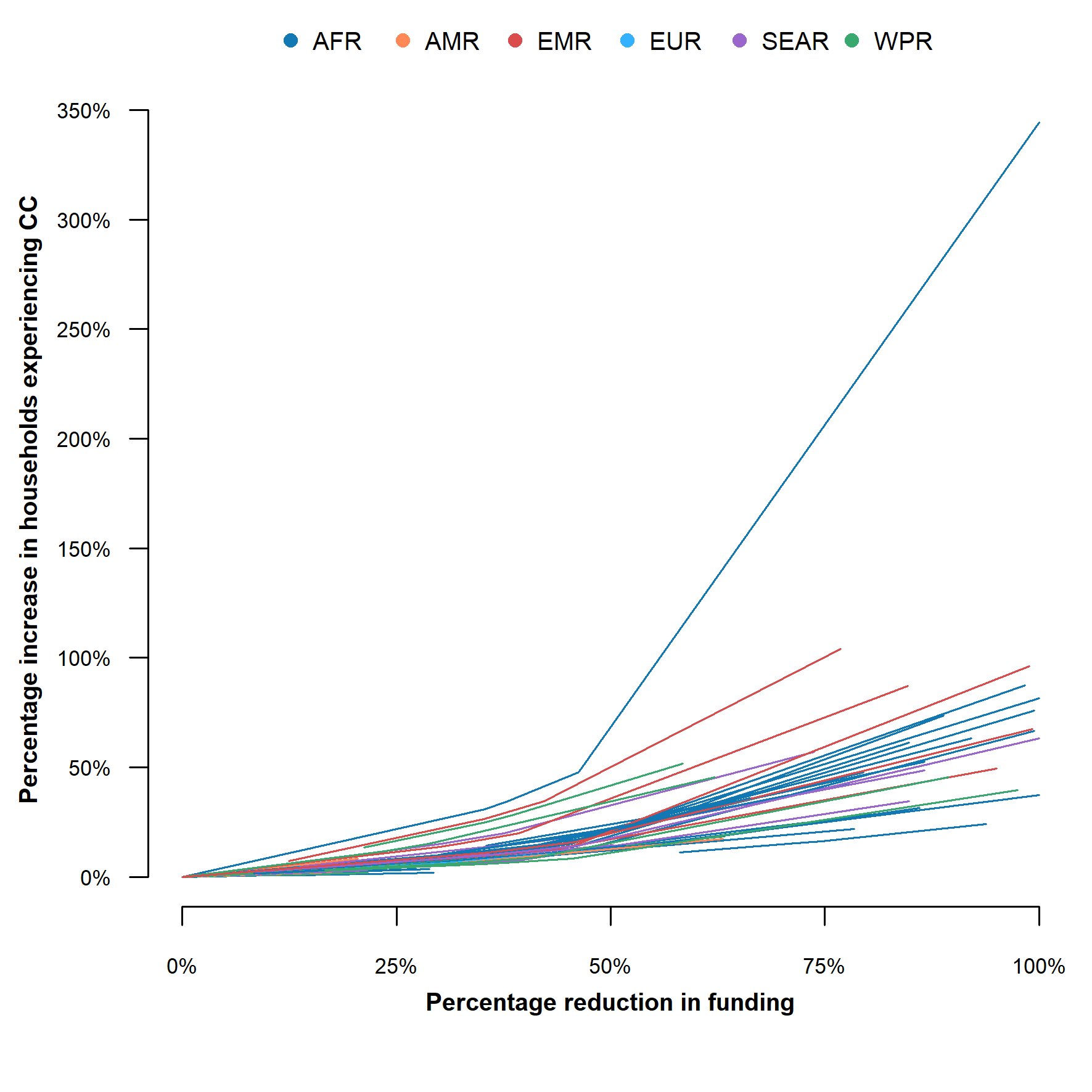


Each line represents analytic results for an individual country, based on the range of funding cut scenarios examined in the analysis.

# Supplementary Figure D. Distribution of the number of TB-affected households experiencing catastrophic costs over 2025–2050 given (1) the termination of funding from USAID + reductions in contributions to GF from the USA alone; and (2) the termination of funding from USAID + termination of all contributions to GF from the USA alone, across all modelled strata, ordered by household income.


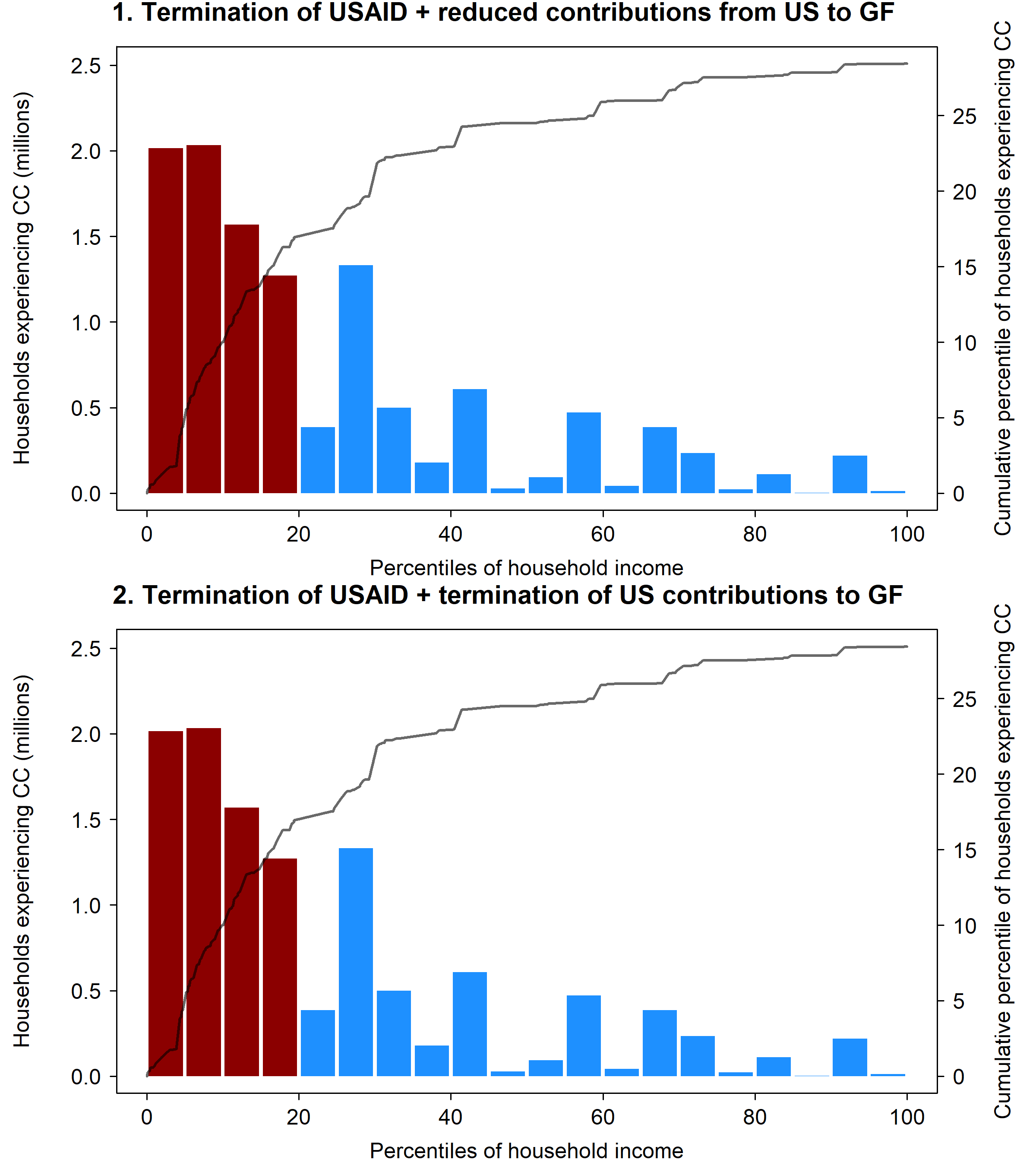


Bars defined by left-hand side y-axis; lines defined by right-hand side y-axis. Ordering of population by household income based on average 2021 per capita GDP in purchasing power parity (PPP) dollars, for each modelled stratum (395 total strata). Bars shaded red indicate the poorest 20% of modelled population by PPP GDP per capita; bars shaded blue indicate the upper 80% of the modelled population. GDP: gross domestic product; GF: the Global Fund to Fight AIDS, TB and Malaria; TB: tuberculosis; USAID: the United States Agency of International Development.

# Supplementary Table B. Costs borne by TB-affected households and number of households with catastrophic costs (in millions), assuming costs for untreated cases are 0.5x treated cases.

| **Scenario** | **Increased patient direct medical costs** | **Increased patient direct non–medical costs** | **Increased patient indirect costs** | **Increased patient total costs** | **Increased cases of catastrophic costs** |
| --- | --- | --- | --- | --- | --- |
| Termination of USAID | 0.5 (0.3–0.8) | 1.2 (0.6–1.7) | 1.7 (1.0–2.5) | 3.4 (2.1–4.6) | 2.3 (1.9–2.7) |
|  | *1.2% (0.7–1.7%)* | *1.1% (0.6–1.7%)* | *1.4% (0.8–2.0%)* | *1.2% (0.7–1.7%)* | *3.2% (2.6–3.8%)* |
| Termination of USAID and reduced Global Fund contributions from the USA | 1.5 (0.9–2.3) | 3.6 (1.8–5.3) | 4.7 (2.5–7.0) | 9.9 (5.4–14.0) | 6.9 (5.6–8.1) |
|  | *3.5% (2.0–5.2%)* | *3.4% (1.8–5.3%)* | *3.9% (2.2–5.5%)* | *3.6% (2.0–5.3%)* | *9.4% (7.5–11.1%)* |
| Termination of USAID and termination of Global Fund contributions from the USA | 1.6 (0.9–2.5) | 3.8 (1.9–5.6) | 4.9 (2.6–7.4) | 10.4 (5.6–14.7) | 7.3 (5.9–8.6) |
|  | *3.7% (2.1–5.4%)* | *3.6% (1.9–5.6%)* | *4.1% (2.3–5.9%)* | *3.8% (2.0–5.6%)* | *10.0% (7.9–11.9%)* |
| Termination of USAID and reduced Global Fund contributions from donor countries contributing 1% or more to the budget | 1.9 (1.1–2.9) | 4.5 (2.1–6.7) | 5.7 (2.9–8.6) | 12.1 (6.2–17.6) | 8.7 (7.0–10.3) |
|  | *4.3% (2.4–6.4%)* | *4.2% (2.1–6.7%)* | *4.7% (2.5–6.9%)* | *4.4% (2.3–6.6%)* | *11.9% (9.4–14.3%)* |
| Termination of all external TB funding | 3.7 (1.4–6.5) | 8.8 (2.6–15.5) | 10.1 (3.6–17.3) | 22.5 (7.4–37.7) | 24.1 (18.6–30.1) |
|  | *8.3% (3.2–14.4%)* | *8.2% (2.8–14.7%)* | *8.3% (3.2–14.6%)* | *8.3% (2.7–14.4%)* | *33.1% (25.2–42.4%)* |

Note: Estimates include 79 low- and middle-income countries analyzed compared to the baseline scenario. Values in italicized text represent percentage changes compared to the baseline scenario. Values in parentheses represent equal-tailed 95% credible intervals. Total costs included patient direct medical, direct non-medical, and indirect costs (all undiscounted) over 2025–2050 in 2021 USD. Catastrophic costs are defined as instances where the total patient costs incurred during an episode of TB disease exceeded 20% of total annual household income. GF = Global Fund to Fight AIDS, TB and Malaria; TB = tuberculosis; USAID = United States Agency of International Development.

# Supplementary Table C. Costs borne by TB-affected households and number of households with catastrophic costs (in millions), assuming costs for untreated cases are 1.5x treated cases.

| **Scenario** | **Increased patient direct medical costs** | **Increased patient direct non–medical costs** | **Increased patient indirect costs** | **Increased patient total costs** | **Increased cases of catastrophic costs** |
| --- | --- | --- | --- | --- | --- |
| Termination of USAID | 1.8 (1.3–2.4) | 4.3 (3.3–5.2) | 5.5 (4.4–6.8) | 11.7 (10.1–13.4) | 4.8 (3.9–5.6) |
|  | *3.2% (2.6–4.0%)* | *3.1% (2.4–3.8%)* | *3.8% (3.2–4.4%)* | *3.4% (2.9–4.0%)* | *3.0% (2.4–3.5%)* |
| Termination of USAID and reduced Global Fund contributions from the USA | 5.7 (4.1–7.2) | 13.3 (10.5–16.1) | 16.2 (13.0–19.6) | 35.3 (29.9–40.5) | 14.3 (11.4–16.9) |
|  | *10.2% (7.8–12.5%)* | *9.7% (7.3–12.0%)* | *11.2% (9.4–12.9%)* | *10.4% (8.7–12.1%)* | *8.9% (7.0–10.8%)* |
| Termination of USAID and termination of Global Fund contributions from the USA | 6.1 (4.4–7.7) | 14.2 (11.2–17.2) | 17.2 (13.8–20.9) | 37.5 (31.8–43.2) | 15.1 (12–18.1) |
|  | *10.8% (8.3–13.4%)* | *10.3% (7.8–12.7%)* | *11.9% (10.0–13.7%)* | *11.1% (9.2–12.9%)* | *9.4% (7.4–11.5%)* |
| Termination of USAID and reduced Global Fund contributions from donor countries contributing 1% or more to the budget | 7.4 (5.3–9.5) | 17.1 (13.5–20.7) | 20.7 (16.5–25.2) | 45.2 (38.2–52.1) | 18.1 (14.3–21.8) |
|  | *13.1% (10.1–16.2%)* | *12.5% (9.4–15.3%)* | *14.3% (12.0–16.5%)* | *13.3% (11.1–15.6%)* | *11.3% (8.8–13.9%)* |
| Termination of all external TB funding | 22.8 (16.1–29.7) | 51.4 (38.4–64.6) | 62.7 (48.1–78.5) | 136.8 (112.3–161.2) | 50.1 (38.0–63.0) |
|  | *40.5% (30.8–50.7%)* | *37.4% (27–47.9%)* | *43.3% (35.1–52.4%)* | *40.4% (32.1–49.4%)* | *31.2% (23.7–40.6%)* |

Note: Estimates include 79 low- and middle-income countries analyzed compared to the baseline scenario. Values in italicized text represent percentage changes compared to the baseline scenario. Values in parentheses represent equal-tailed 95% credible intervals. Total costs included patient direct medical, direct non-medical, and indirect costs (all undiscounted) over 2025–2050 in 2021 USD. Catastrophic costs are defined as instances where the total patient costs incurred during an episode of TB disease exceeded 20% of total annual household income. GF = Global Fund to Fight AIDS, TB and Malaria; TB = tuberculosis; USAID = United States Agency of International Development.

# Supplementary Table D. Number of households with catastrophic costs (in millions), assuming thresholds of 10%, 20% (base-case), and 25%.

| **Scenario** | **10% threshold** | **20% threshold (base-case)** | **25% threshold** |
| --- | --- | --- | --- |
| Termination of USAID | 5.4 (4.4–6.3) | 3.9 (3.2–4.6) | 3.4 (2.8–4.0) |
|  | *2.9% (2.4–3.5%)* | *3.1% (2.5–3.6%)* | *3.1% (2.5–3.7%)* |
| Termination of USAID and reduced Global Fund contributions from the USA | 16.0 (12.7–19.1) | 11.5 (9.2–13.6) | 9.9 (8.0–11.8) |
|  | *8.7% (6.9–10.7%)* | *9.1% (7.2–11.0%)* | *9.2% (7.3–11.1%)* |
| Termination of USAID and termination of Global Fund contributions from the USA | 17.0 (13.5–20.4) | 12.2 (9.8–14.5) | 10.6 (8.5–12.5) |
|  | *9.3% (7.3–11.4%)* | *9.6% (7.6–11.7%)* | *9.7% (7.7–11.8%)* |
| Termination of USAID and reduced Global Fund contributions from donor countries contributing 1% or more to the budget | 20.3 (16.1–24.6) | 14.6 (11.6–17.6) | 12.6 (10.1–15.1) |
|  | *11.1% (8.7–13.7%)* | *11.5% (9.0–14.1%)* | *11.7% (9.2–14.2%)* |
| Termination of all external TB funding | 56.2 (42.6–70.9) | 40.5 (30.9–50.7) | 35.0 (26.7–43.8) |
|  | *30.7% (23.3–40.0%)* | *31.9% (24.2–41.3%)* | *32.3% (24.6–41.6%)* |

Note: Estimates include 79 low- and middle-income countries analyzed compared to the baseline scenario. Values in italicized text represent percentage changes compared to the baseline scenario. Values in parentheses represent equal-tailed 95% credible intervals. Catastrophic costs are defined as instances where the total patient costs incurred during an episode of TB disease exceeded 20% of total annual household income. GF = Global Fund to Fight AIDS, TB and Malaria; TB = tuberculosis; USAID = United States Agency of International Development.
